# Conduction Mechanism and Magnetic Property of Ag-Doped LaFeO_3_ Nanofibers

**DOI:** 10.3390/molecules31071174

**Published:** 2026-04-02

**Authors:** Chao Song, Jiayue Xu, Hanqiong Luo, Quanli Hu

**Affiliations:** Inner Mongolia Key Lab of Solid State Chemistry for Battery, Inner Mongolia Engineering Research Center of Lithium-Sulfur Battery Energy Storage, College of Chemistry and Materials Science, Inner Mongolia Minzu University, Tongliao 028000, China; sc199639@outlook.com (C.S.); xjy20030723@outlook.com (J.X.); luohanqiong@outlook.com (H.L.)

**Keywords:** conduction mechanism, LaFeO_3_, magnetic, nanofiber

## Abstract

LaFeO_3_ nanofibers and Ag-doped LaFeO_3_ nanofibers were fabricated via an approach combining electrospinning with calcination. Their crystal structures, micro-morphologies, and chemical compositions were determined by X-ray diffraction, scanning electron microscopy, transmission electron microscopy, X-ray photoelectron spectroscopy, Raman spectroscopy, and Fourier-transform infrared spectroscopy. In addition, the conduction mechanisms and magnetic properties of the two samples were investigated using a semiconductor analyzer and a vibrating sample magnetometer. Rietveld refined X-ray diffraction analyses confirmed the orthorhombic structure. The two samples showed a nanofibrous structure. For Ag-doped LaFeO_3_, the conduction was dominated by the ohmic conduction mechanism in a low-resistance state, while it was governed by space-charge-limited current conduction in a high-resistance state. It also showed a high on/off ratio of 3.6 × 10^3^. The coercivity and remanence values of Ag-doped LaFeO_3_ were 200 Oe and 0.000404 emu g^−1^. This, thus, indicates the considerable application potential of Ag-doped LaFeO_3_ for resistive random-access memory devices and magnetoresistive random-access memory devices.

## 1. Introduction

ABO_3_-type perovskites have attracted tremendous research attention on account of their exceptional magnetic and electronic functionalities [1]. The perovskite crystal structure consists of a three-dimensional framework of corner-sharing BO_6_ octahedra, where A-site cations are situated in the cuboctahedral voids [2]. By varying the constituent elements within the ABO_3_ framework, these materials can be engineered for magnetic functional, electronic and energy applications [1,3]. Lanthanum ferrite (LaFeO_3_) is a typical lanthanum-based perovskite material and possesses stable electronic structure, efficient electron transport, p-type conductivity, low resistance and favorable photocatalytic activity. These merits have enabled the application of LaFeO_3_ in fields such as sensor technology, environmental remediation, and energy development [2,4,5,6]. In the ideal perovskite structure, the A-site La cation exhibits a coordination number of 12. For orthorhombically distorted LaFeO_3_, structural distortion reduces the coordination environment of La to approximately 8 [1,2]. The mixed ionic–electronic conductor that exhibits reversible redox behavior renders LaFeO_3_ a highly attractive candidate for resistive switching applications [7,8]. LaFeO_3_ is a representative G-type antiferromagnetic material. The super-exchange and Dzyaloshinsky–Moriya interactions between Fe ions induce the formation of a canted antiferromagnetic order in LaFeO_3_ [1]. The conditions for ferroelectricity and magnetism are mutually exclusive, apart from a small number of multiferroic materials such as BiFeO_3_ and LaFeO_3_ [8]. It is reported that the surface termination of perovskite oxides is typically dominated by inert AO planes, as opposed to the reactive B-site centers, which leads to the passivation of surface reactivity and related functionalities. By engineering A-site metal ion vacancies within the perovskite lattice, the oxidation state of B-site cations and the density of oxygen vacancies can be efficiently tuned. Additionally, this defect engineering strategy can induce lattice distortion in the perovskite, thereby facilitating the generation of localized regions with high electron density [9]. A-site lattice imperfections and the induced oxygen vacancies synergistically enhance surface reaction activity, and furnish optimal nucleation loci for the deposition of Ag and analogous metal nanoparticles, facilitating the creation of strong metal–support interactions and effective interfacial Schottky junctions. Consequently, A-site defect engineering in LaFeO_3_ enables high-performance photocatalyst nanocomposites for CO_2_ reduction [9]. Noble metal doping is an effective approach to enhance the practical application performance of LaFeO_3_ [6]. Silver ions can intercalate into the crystal lattice of LaFeO_3_, introducing reactive sites and enhancing the efficiency of charge carrier separation and utilization [6,9]. Ag-doped LaFeO_3_ shows great potential application in ethanol detection, photocatalysts for CO_2_ conversion to CH_3_OH, and gas sensors [2,6,9,10]. LaFeO_3_ and its doped variants can be prepared using several techniques, including electrospinning, hydrothermal synthesis, solid-state reaction, and combustion synthesis [11,12,13,14]. Electrospinning is a facile method to controllably prepare continuous one-dimensional nanofibers (NFs) with a large aspect ratio, high specific surface area, and uniform porous structure [15]. These features are highly favorable for charge transport and interface-related properties in resistive switching and magnetic applications. This method allows homogeneous doping of Ag ions in the LaFeO_3_ lattice during the nanofiber-forming process, ensuring good compositional uniformity and structural stability.

This study aims to synthesize one-dimensional Ag-doped LaFeO_3_ NFs via electrospinning; systematically investigate the structural evolution, conduction behavior, and magnetic properties; and reveal the influence of Ag doping on the microstructure and multifunctional performance of LaFeO_3_-based NFs. Via electrospinning combined with calcination at high temperature, pure and Ag-doped LaFeO_3_ NFs were successfully synthesized. The crystalline structure, microstructural features, and chemical composition of the fabricated samples were systematically characterized. This study confirms that the Ag-doped LaFeO_3_ NFs show significant potential for resistive random-access memory and magnetoresistive random-access memory.

## 2. Results and Discussion

### 2.1. Structural Investigation

**Figure 1:**
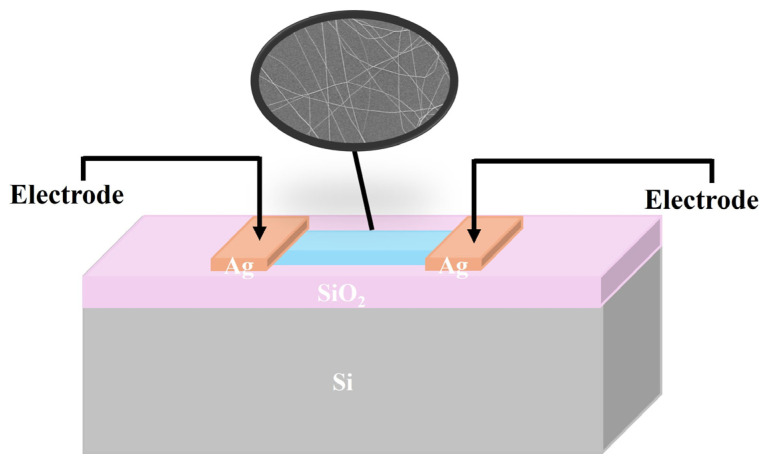
The schematic illustration of Ag/Ag_0.025_LaFeO_3_ NFs/Ag devices.

Figure 1 is the schematic illustration of the fabricated devices.

**Figure 2:**
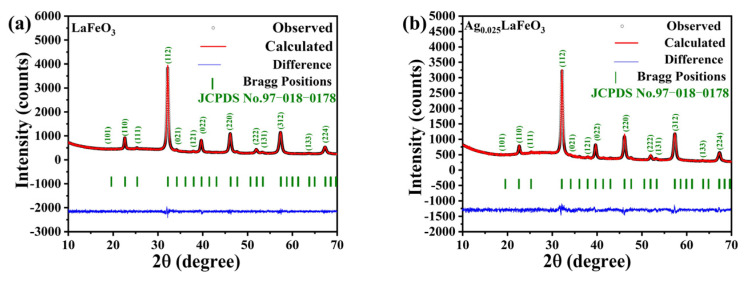
Rietveld refined X-ray diffraction patterns of (**a**) LaFeO_3_ and (**b**) Ag_0.025_LaFeO_3_.

Rietveld refinement of the XRD data was used to determine the lattice parameters of LaFeO_3_ and Ag_0.025_LaFeO_3_. As shown in Figure 2, XRD patterns exhibited sharp and narrow peaks with high intensity, which was indicative of good crystallinity in the synthesized LaFeO_3_ and Ag_0.025_LaFeO_3_. According to JCPDS No. 97-018-0178, it confirmed the orthorhombic crystal structure with the *Pbnm(62)* space group, and no secondary or impurity phases were observed. XRD results proved the complete dissolution of Ag^+^ into the LaFeO_3_ lattice without impurity formation. The ionic-radius values of Ag^+^, La^3+^ and Fe^3+^ are 0.115 nm, 0.103 nm and 0064 nm, respectively. Given the similar ionic radii of Ag^+^ and La^3+^, Ag^+^ ions can easily occupy the La^3+^ positions in the LaFeO_3_ perovskite structure [6]. The larger ionic radius of Ag^+^ than that of Fe^3+^ may lead to an increase in the Fe-O bond length, thereby causing the lattice to expand along the *b*-axis. Therefore, the introduction of Ag ions in LaFeO_3_ led to slight lattice expansion. Furthermore, the goodness-of-fit (GOF) values obtained from the Rietveld refinement for LaFeO_3_ and Ag_0.025_LaFeO_3_ were less than 2, which confirmed the high accuracy of the refinement results. The lattice parameters of LaFeO_3_ and Ag_0.025_LaFeO_3_ were summarized in Table 1.

### 2.2. Morphological Analyses

**Figure 3:**
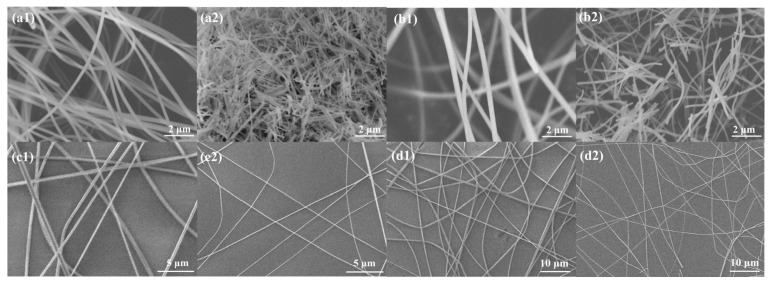
SEM images of (**a1**) La(NO_3_)_3_-FeAc_2_/PVP precursor, (**a2**) LaFeO_3_ NFs, (**b1**) La(NO_3_)_3_-FeAc_2_-AgAc/PVP precursor, (**b2**) Ag_0.025_LaFeO_3_ NFs, (**c1**) La(NO_3_)_3_-FeAc_2_/PVP precursor on SiO_2_/Si, (**c2**) LaFeO_3_ NFs on SiO_2_/Si, (**d1**) La(NO_3_)_3_-FeAc_2_-AgAc/PVP precursor on SiO_2_/Si, and (**d2**) Ag_0.025_LaFeO_3_ NFs on SiO_2_/Si.

To characterize the morphological and structural features, the LaFeO_3_ and Ag_0.025_LaFeO_3_ samples were systematically investigated via SEM measurement. As shown in Figure 3(a1,b1), La(NO_3_)_3_-FeAc_2_/PVP and La(NO_3_)_3_-FeAc_2_-AgAc/PVP precursors exhibited a smooth nanofibrous morphology, and the electrospun precursor NFs were intertwined with each other. The NFs were relatively uniformly distributed without obvious agglomeration or clumping, featuring a narrow diameter distribution and regular morphology, with diameters ranging from approximately 240 to 380 nm. The surfaces of the precursor NFs were smooth and free of pores or protrusions, which was attributed to the rapid volatilization of DMF during the electrospinning process and the uniform solidification and formation of PVP and metal salt precursors. According to Figure 3(a2,b2), high-temperature calcination induced a profound morphological evolution of the precursor NFs, featuring shortened length, diminished diameter size, and distinctly enhanced surface roughness. This phenomenon was mainly attributed to the decomposition of PVP and the decomposition and crystallization shrinkage of La(NO_3_)_3_, FeAc_2_, and AgAc during the calcination process. Additionally, the NFs retained a narrow diameter distribution after calcination. Figure 3(c1,d1) presented La(NO_3_)_3_-FeAc_2_/PVP and La(NO_3_)_3_-FeAc_2_-AgAc/PVP precursors on SiO_2_/Si substrate. These precursor NFs exhibited a continuous and interconnected network structure. According to Figure 3(c2,d2), these network structures were still maintained even after high-temperature calcination treatment, and most nanofibers retained a continuous morphology.

**Figure 4:**
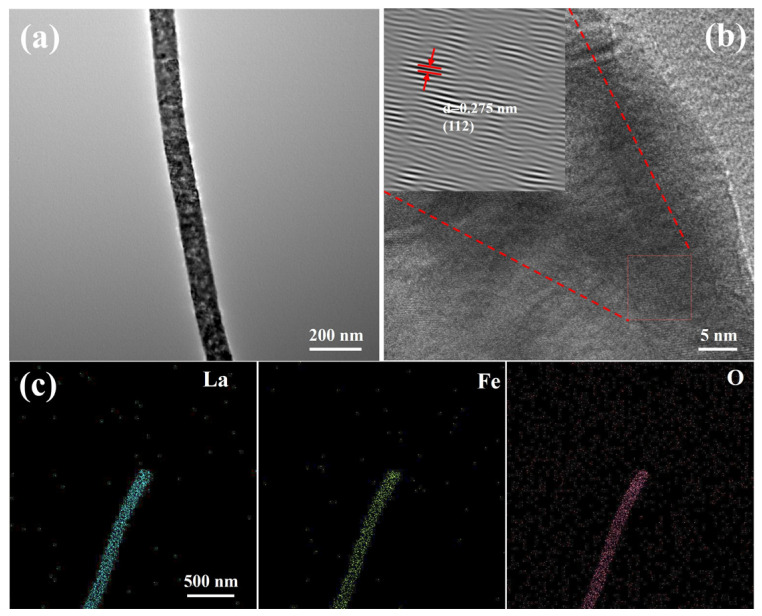
(**a**) TEM image, (**b**) HRTEM image, and (**c**) elemental mapping images of LaFeO_3_ NF. Inset is the lattice fringe image.

**Figure 5:**
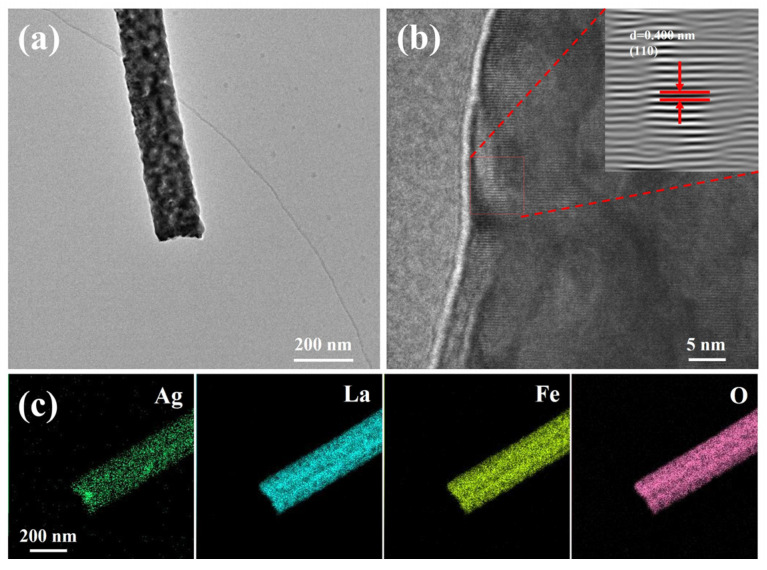
(**a**) TEM image, (**b**) HRTEM image, and (**c**) elemental mapping images of Ag_0.025_LaFeO_3_ NF. Inset is the lattice fringe image.

To further investigate the microstructural morphology and elemental distribution characteristics of the synthesized LaFeO_3_ and Ag_0.025_LaFeO_3_ NFs, TEM characterization was conducted on the two samples. Based on Figure 4a and Figure 5a, LaFeO_3_ and Ag_0.025_LaFeO_3_ exhibited nanofibrous structures that were in good agreement with those obtained from SEM characterization. The diameter values of LaFeO_3_ and Ag_0.025_LaFeO_3_ were about 100 and 160 nm, respectively. According to Figure 4b and Figure 5b, clear lattice fringes were visible in the high-resolution transmission electron microscopy (HRTEM) micrographs, where the lattice spacing of 0.275 and 0.400 nm matched well with the (112) and (110) crystal planes for LaFeO_3_ and Ag_0.025_LaFeO_3_ NFs. As shown in Figure 4c and Figure 5c, the elemental mapping images clearly demonstrated the homogeneous distribution of La, Fe, and O elements in the LaFeO_3_, as well as the La, Ag, Fe, and O elements in Ag_0.025_LaFeO_3_ NFs. Also, it further confirmed that the doped Ag ions were uniformly dispersed without obvious aggregation. The relatively weaker Ag signal observed at grain boundaries can be ascribed to the inhomogeneous incorporation of Ag dopant within the LaFeO_3_ lattice. The Ag species preferentially entered the perovskite lattice sites instead of accumulating as dense agglomerates on the surface.

### 2.3. XPS Analysis

**Figure 6:**
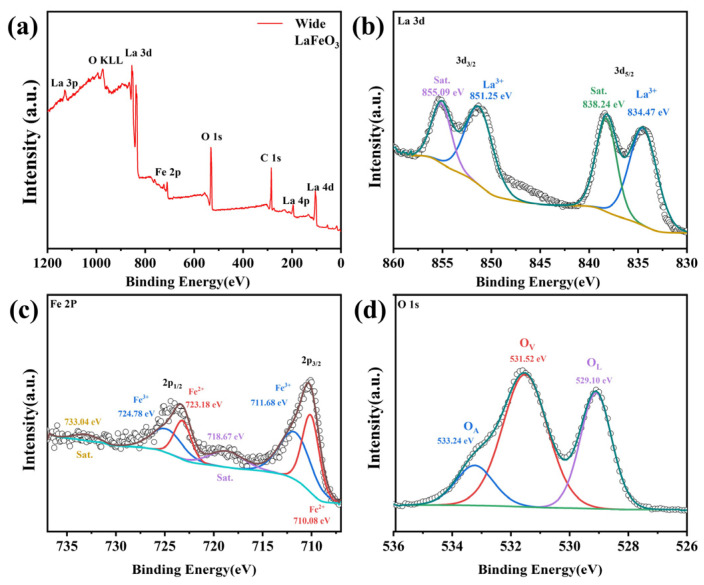
(**a**) Survey scan, (**b**) La 3d, (**c**) Fe 2p, and (**d**) O 1s XPS profiles of LaFeO_3_ NFs.

**Figure 7:**
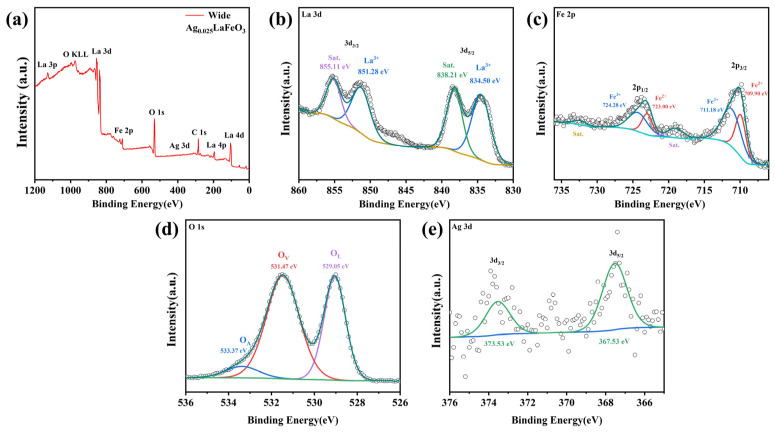
(**a**) Survey scan, (**b**) La 3d, (**c**) Fe 2p, (**d**) O 1s and (**e**) Ag 3d XPS profiles of Ag_0.025_LaFeO_3_ NFs.

The chemical states and electronic structures of the elements contained in LaFeO_3_ and Ag_0.025_LaFeO_3_ NFs were investigated by means of XPS measurements. As illustrated in Figure 6a and Figure 7a, the full survey scan spectra confirmed the presence of La, Fe, and O in LaFeO_3_, as well as La, Fe, O, and Ag in Ag_0.025_LaFeO_3_. Adventitious carbon contamination was signaled by the observed C 1s peak, and this contamination was tentatively attributed to the substrate or instrumental components. High-resolution XPS spectra for La 3d, Fe 2p, O 1s, and Ag 3d are illustrated in Figure 6 and Figure 7. A good consistency was observed between the elemental mapping and XPS results. In the high-resolution La 3d XPS spectra of LaFeO_3_ and Ag_0.025_LaFeO_3_, four well-defined peaks can be observed in Figure 6b and Figure 7b. Two distinct peaks centered at roughly 834 and 851 eV were assigned to La 3d_5/2_ and La 3d_3/2_, respectively. The binding energy gap between these two peaks was approximately 17 eV, indicating a definitive hallmark of the La^3+^ valence state [16]. Spin–orbit interaction gave rise to a satellite peak at about 838 eV, while electron transfer between the O 2p valence band and La 4f energy level was responsible for the satellite peak at roughly 855 eV [17]. The Fe 2p spectra of LaFeO_3_ and Ag_0.025_LaFeO_3_ NFs, as shown in Figure 6c and Figure 7c, exhibited two main spin–orbit split regions, namely Fe 2p_3/2_ and Fe 2p_1/2_. The sub-peaks at 711 eV and 724 eV were attributed to Fe^3+^, and those at 710 eV and 723 eV were ascribed to Fe^2+^ [16]. Trivalent Fe was identified as the dominant valence state of the Fe component. Figure 6d and Figure 7d show the high-resolution XPS spectrum of the O 1s orbital for LaFeO_3_ and Ag_0.025_LaFeO_3_ NFs. Through peak-fitting analysis, the O 1s spectra can be deconvoluted into three characteristic components, namely lattice oxygen (O^2−^, denoted as O_L_) at a binding energy of 529 eV, surface-adsorbed oxygen or oxygen vacancy defects (O_2_^2−^/O^−^, denoted as O_V_) at 531 eV, and surface hydroxyl groups or adsorbed water (OH^−^/H_2_O, denoted as O_A_) [6]. Figure 7e presents the high-resolution XPS spectrum of the Ag 3d orbital for Ag_0.025_LaFeO_3_ NFs. The characteristic peaks can be observed at the binding energies of 367.53 eV (Ag 3d_5/2_) and 373.53 eV (Ag 3d_3/2_), which confirms the presence of Ag^+^ in the Ag_0.025_LaFeO_3_ sample [18]. A comparison of the high-resolution O 1s spectra between the LaFeO_3_ and Ag_0.025_LaFeO_3_ revealed a slight leftward shift in the O_L_ and O_V_ peak positions for Ag_0.025_LaFeO_3_, demonstrating that Ag doping introduced lattice defects in LaFeO_3_. When Ag^+^ is doped into the A-site of LaFeO_3_ to substitute La^3+^, a charge imbalance is induced. To maintain the electrical neutrality of the perovskite lattice, the dominant charge compensation mechanisms include the reduction of partial Fe^3+^ to Fe^2+^ in the B-site and the formation of oxygen vacancies. Both EDS and XPS are recognized as semi-quantitative techniques. Noticeable modifications in magnetic behavior and carrier transport characteristics are observed upon Ag doping.

### 2.4. Raman Analyses

**Figure 8:**
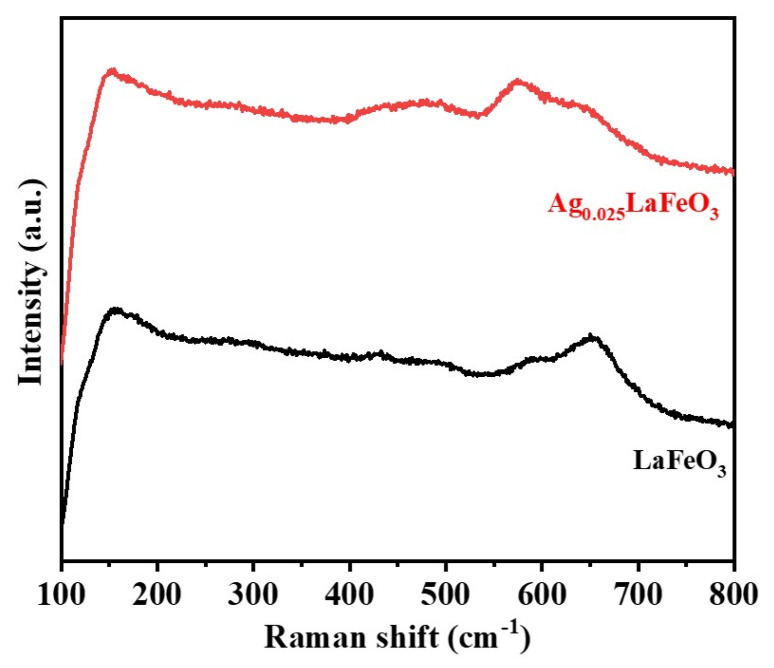
Raman spectra of LaFeO_3_ and Ag_0.025_LaFeO_3_ NFs.

To investigate the chemical bonding states of LaFeO_3_ and Ag_0.025_LaFeO_3_, Raman spectroscopy characterization was performed. Both samples were tested at room temperature using a Raman spectrometer with a laser excitation wavelength of 633 nm, and the measurements were conducted in the range of 100–800 cm^−1^. The results are presented in Figure 8. An obvious peak at 154 cm^−1^ corresponded to the vibration of La-O [19]. The peak located in 258–300 cm^−1^ was assigned to the tilting vibration mode of FeO_6_, which enabled the determination of the degree of lattice distortion and tilting [19]. The bending vibration mode of FeO_6_ at 430 cm^−1^ can be seen. The single peak in the range of 612–650 cm^−1^ corresponded to the oxygen stretching vibration mode, and the strong peak in this region was generally associated with oxygen vacancies [20,21]. The changes in Raman peak intensity at 571 cm^−1^ and 494 cm^−1^ could originate from the replacement of La^3+^ by Ag^+^ [19].

### 2.5. FTIR Analyses

**Figure 9:**
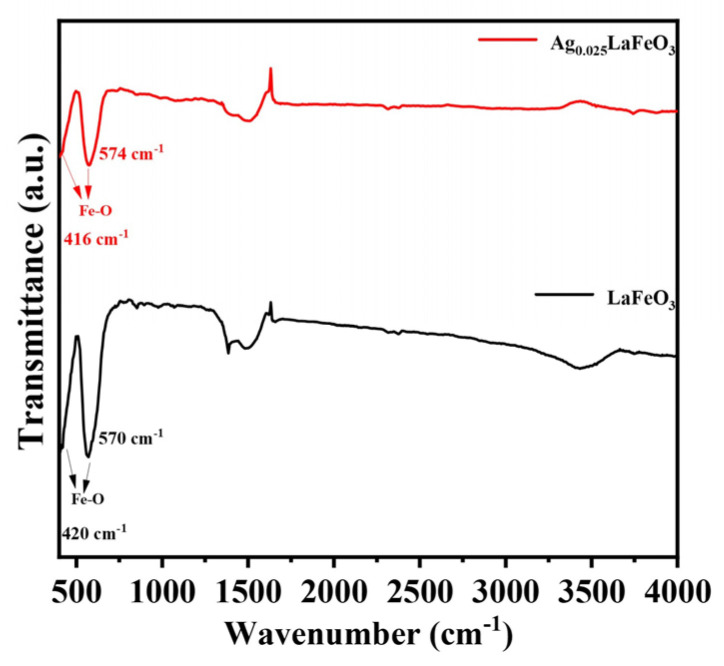
FTIR spectra of LaFeO_3_ and Ag_0.025_LaFeO_3_ NFs.

Fourier-transform infrared spectra of LaFeO_3_ and Ag_0.025_LaFeO_3_ are shown in Figure 9. The data were recorded in the wavenumber range from 400 to 4000 cm^−1^. The characteristic peaks located at about 420 and 570 cm^−1^ originated from the stretching vibration of Fe-O bonds [2,22]. Weak absorption signals near 3500 cm^−1^ and 1500 cm^−1^ corresponded to O-H stretching and water bending vibrations, respectively, originating from moisture adsorbed on the sample surface [2]. The peak at 1380 cm^−1^ was ascribed to monodentate carbonate species [2].

### 2.6. Conduction Mechanisms

**Figure 10:**
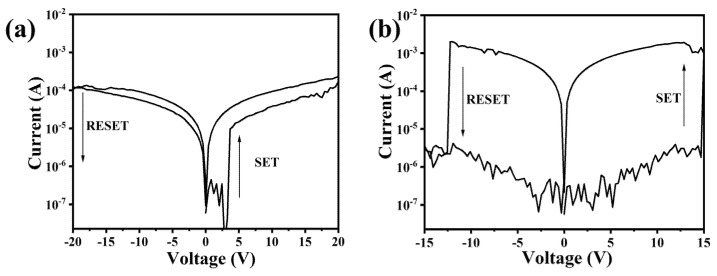
I–V curves (**a**) Ag/LaFeO_3_ NFs/Ag and (**b**) Ag/Ag_0.025_LaFeO_3_ NFs/Ag.

Ag/LaFeO_3_ NFs/Ag and Ag/Ag_0.025_LaFeO_3_ NFs/Ag devices based on LaFeO_3_ and Ag_0.025_LaFeO_3_ NFs were prepared, with the aim of studying their electrical conduction mechanisms. According to the I–V curves as shown in Figure 10, two devices presented bipolar resistive switching performance. The resistance changed from a high-resistance state (HRS) to a low-resistance state (LRS) at high positive voltage, while it changed from LRS to HRS at high negative voltage. A substantially low on/off ratio was observed in the LaFeO_3_ NF dielectric layer device relative to the Ag_0.025_LaFeO_3_ NF counterpart. The on/off ratio at +5 V of Ag/Ag_0.025_LaFeO_3_ NFs/Ag was 3.6 × 10^3^, while that of Ag/LaFeO_3_ NFs/Ag was 3.4. The radius and valence state of Ag^+^ differed from those of La^3+^ and Fe^3+^ in the LaFeO_3_ lattice. To maintain electrical neutrality after doping, the lattice generated more oxygen vacancies as charge compensation sites. The increased oxygen vacancy concentration within the Ag_0.025_LaFeO_3_ NFs was induced by Ag doping, which benefited the formation of conductive filaments and enhanced the migration ability of oxygen vacancies under applied voltage. The high oxygen vacancy concentration facilitated the formation of conductive filaments during the SET process and promoted complete rupture during the RESET process, thereby widening the resistance difference between the HRS and LRS and improving the on/off ratio [23]. The SET and RESET voltage values of Ag_0.025_LaFeO_3_ NFs were 14.7 and −12.3 V, while those of LaFeO_3_ NFs were 3.2 and −18.1 V. During the SET process, it was necessary not only to drive the migration of oxygen vacancies but also to facilitate the injection of Ag ions from the electrodes into Ag_0.025_LaFeO_3_ NFs. A relatively high voltage was thus required to enable the effective migration of Ag ions and the formation of conductive filaments. In the RESET process, the negative voltage should not only oxidize the oxygen vacancies but also drive the deintercalation of Ag ions from the filaments and their migration back to the electrodes. It also required a high RESET voltage [24,25].

**Figure 11:**
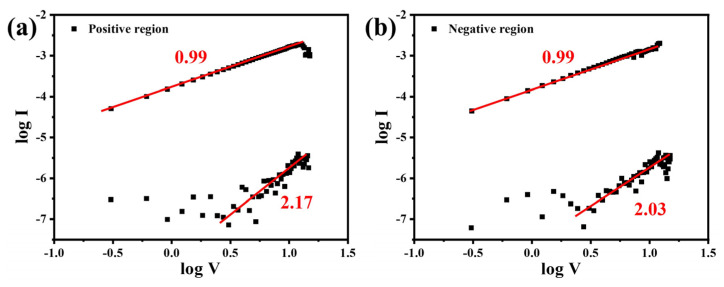
Log–log I–V curves of Ag/Ag_0.025_LaFeO_3_ NFs/Ag in (**a**) positive-voltage region and (**b**) negative-voltage region.

The double-logarithmic I–V curves were plotted to analyze the charge transport behavior of Ag_0.025_LaFeO_3_ NFs. Figure 11 shows the log–log I–V curves of Ag/Ag_0.025_LaFeO_3_ NFs/Ag in the positive- and negative-voltage regions. As shown in Figure 11a, they exhibited a distinct linear region before SET, indicating the ohmic conduction mechanism. After SET, the slope of 2.17 corresponded to the space-charge-limited current (SCLC) conduction mechanism, confirming that Ag doping introduced oxygen vacancies as carrier trap sites and that the Ag/Ag_0.025_LaFeO_3_ NFs/Ag device operated primarily via the SCLC mechanism [26]. As shown in Figure 11b, before RESET, the slope of 0.99 aligned with the ohmic conduction mechanism, demonstrating that carrier transport in this region was dominated by ohmic conduction. After RESET, the slope of 2.03 corresponded to the trap-filled SCLC conduction mechanism, verifying that Ag doping introduced oxygen vacancies as carrier trap sites and that the device was governed by the SCLC mechanism. The presence of traps further suppressed the current in the HRS, while the current in the LRS was amplified due to the high conductivity of conductive filaments, thus increasing the on/off ratio.

Oxygen vacancies were randomly distributed in Ag_0.025_LaFeO_3_ NFs at the initial state. An abundance of defect traps originating from oxygen vacancies dominated carrier capture, showing typical trap-controlled SCLC characteristics. When a positive voltage was applied to the device, a substantial number of oxygen vacancies were generated, which aggregated to form conductive filaments under the mediation of Ag ions. The resistance switched from the HRS to the LRS. Localized Joule heating played a critical role in this process. This thermal effect, mainly triggered by electron injection, led to the disappearance of oxygen ions at the interface between the electrode and the switching layer [27]. This phenomenon induced the formation of conductive filaments in Ag_0.025_LaFeO_3_ NFs, accompanied by the accumulation of oxygen vacancies at the interface, thus forming protruding filamentary pathways. These filamentary pathways then established serial interconnections, driving the device to shift from the HRS to the LRS, which marked the accomplishment of the SET procedure. During the RESET process, a negative voltage was applied to the device, which caused the rupture of oxygen vacancy-mediated conductive filaments, thus switching the device from the LRS to the HRS. During the SET process, the applied positive voltage facilitated the reduction of Fe^3+^ to Fe^2+^ in Ag_0.025_LaFeO_3_ NFs via the mediation of oxygen vacancies. Conversely, in the RESET process, the application of a negative voltage led to the dissolution of conductive filaments. As the device switched from the LRS to the HRS, the decrease in oxygen vacancy concentration triggered the re-oxidation of Fe^2+^ to Fe^3+^ [28,29].

### 2.7. Magnetic Property

**Figure 12:**
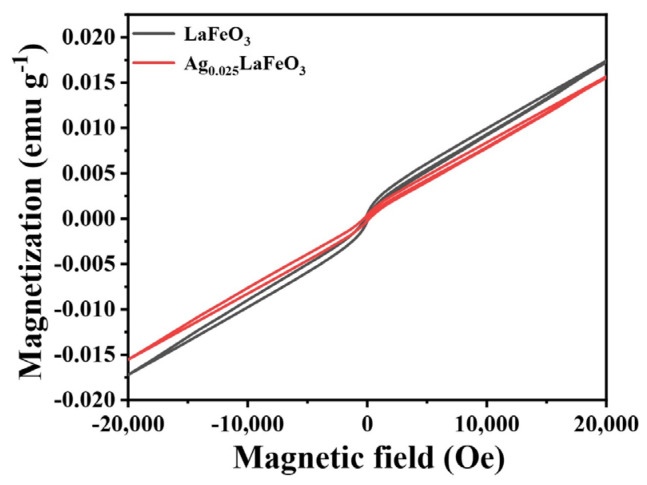
Magnetic hysteresis loops of LaFeO_3_ and Ag_0.025_LaFeO_3_ NFs at 300 K.

The hysteresis loops of LaFeO_3_ and Ag_0.025_LaFeO_3_ NFs measured at 300 K was shown in Figure 12. Under a 20,000/−20,000 Oe external magnetic field, the M-H loops showed no saturation, implying the coexistence of weak ferromagnetism and antiferromagnetism induced by antiferromagnetic spins [29]. The antiferromagnetic structure of LaFeO_3_ and Ag_0.025_LaFeO_3_ stemmed from two coplanar interpenetrating pseudo-rectangular FeO_6_ octahedra, with no magnetic interactions between La^3+^ and Fe^3+^ ions [30]. The pronounced weak ferromagnetism could be attributed to the super-exchange effect of Fe^3+^-O- Fe^3+^. The coercivity (H_c_) and remanence (M_r_) values of LaFeO_3_ were 150 Oe and 0.000494 emu g^−1^, while those of Ag_0.025_LaFeO_3_ were 200 Oe and 0.000404 emu g^−1^. The magnetization value of Ag_0.025_LaFeO_3_ was lower than that of LaFeO_3_. The 4d orbitals of Ag ions were fully occupied, with no unpaired electrons, thus exhibiting diamagnetic behavior. Ag doping induced lattice distortion and changes in the spin canting angle of LaFeO_3_. The radius of Ag^+^ was slightly larger than that of La^3+^, so doping caused lattice expansion and altered the Fe-O-Fe bond angle [31]. Consequently, this affected the strength of antiferromagnetic super-exchange interactions and antisymmetric exchange interactions, leading to a reduction in the spin canting angle, which directly weakened the net weak ferromagnetic moment and thus decreased the remanent magnetization. The introduction of Ag created a charge imbalance and resulted in the formation of oxygen vacancies or the conversion of partial Fe^3+^ to Fe^2+^. An appropriate concentration of oxygen vacancies can disrupt part of the antiferromagnetic coupling pathways and enhance ferromagnetism. The destroyed magnetic ordering or alteration of the contribution of Fe magnetic moments resulted in a decrease in the overall remanent magnetization [32,33].

## 3. Experimental and Methods

Sample fabrication was carried out using lanthanum nitrate hexahydrate (La(NO_3_)_3_·6H_2_O), iron(II) acetate (FeAc_2_), silver acetate (AgAc), polyvinylpyrrolidone (PVP, M_w_ ≈ 1,300,000, K88-96), and N, N-dimethylformamide (DMF). All chemicals were purchased from Aladdin Scientific Corp. Shanghai, China and used as received without undergoing any further purification treatment. La(NO_3_)_3_·6H_2_O, FeAc_2_, and AgAc worked as the precursor for the fabrication of Ag-doped LaFeO_3_, namely Ag_0.025_LaFeO_3_. Ag_0.025_LaFeO_3_ represents the nominal molar ratio of raw materials during synthesis, not a strictly charge-balanced structural formula. The samples were fabricated using an electrospinning technique. Addition of La(NO_3_)_3_·6H_2_O (1 mmol), FeAc_2_ (1 mmol), Ag (0.025 mmol), and PVP (0.5 g) to DMF (4.5 mL) and subsequent 12 h stirring afforded a clear and homogeneous electrospinning solution. Key electrospinning parameters included an applied voltage of 14 kV, a collector roller speed of 140 rpm, a tip-to-collector distance of 30 cm, and an ambient temperature of 40 °C. The as-prepared solution was initially transferred into a disposable syringe, which was subsequently mounted onto the electrospinning apparatus. Meanwhile, cleaned 1.5 cm ×1.5 cm silicon oxide on silicon (SiO_2_/Si) substrates were immobilized on the collector roller using conductive carbon adhesive tape. The precursor solution was then subjected to electrospinning onto the substrates under high-voltage conditions for 2 min, and the silicon wafers were subsequently retrieved. Following the preparation of the electrospun precursor NFs, these were placed in a muffle furnace and calcined at 700 °C for 180 min. The resultant Ag_0.025_LaFeO_3_ samples and the Ag_0.025_LaFeO_3_ NFs on SiO_2_/Si substrates were then obtained after cooling to room temperature. The reference control sample of LaFeO_3_ was prepared by the same method with La(NO_3_)_3_·6H_2_O (1 mmol) and FeAc_2_ (1 mmol).

Magnetron sputtering (JCP5000, Beijing Technol Science Co., Ltd., Beijng, China) was employed to deposit silver electrodes onto the SiO_2_/Si substrates with LaFeO_3_ and Ag_0.025_LaFeO_3_ NFs, followed by the assembly of planar-structured Ag/LaFeO_3_ NFs/Ag and Ag/Ag_0.025_LaFeO_3_ NFs/Ag devices. The silver electrodes were deposited via DC magnetron sputtering using a 99.99%-pure Ag target, which was obtained from ZhongNuo Advanced Material Technology Co., Ltd. Beijing, China. The key sputtering parameters were a sputtering power of 100 W, an argon flow rate of 50 sccm, a sputtering pressure of 0.1 Pa, a background vacuum of about 5 × 10^−5^ Pa, and a sputtering time of 1 min. The substrate was kept at room temperature with no applied bias voltage. The nominal thickness of the silver electrodes was about 500 nm.

The crystal structures of LaFeO_3_ and Ag_0.025_LaFeO_3_ were recorded by an X-ray powder diffractometer (XRD, Rigaku Smartlab, Tokyo, Japan) with Cu Kα radiation (λ = 1.5406 Å) over a 2θ range from 10° to 70°, and then Rietveld refinement was carried out using GSASII revision: 5775 (svn SVN version 5775) software. To characterize the morphological features of electrospun La(NO_3_)_3_-FeAc_2_-AgAc/PVP precursors and resultant Ag_0.025_LaFeO_3_ samples, scanning electron microscopy (SEM, Hitachi S-4800, Hitachinaka, Japan) and transmission electron microscopy (TEM, JEOL JEM F-200, Akishima, Japan) were utilized. The morphology of the Ag_0.025_LaFeO_3_ NFs on SiO_2_/Si substrate was examined by SEM. An energy-dispersive X-ray spectrometer attached to the TEM instrument was utilized for the assessment of elemental distribution profiles. The valence states and chemical compositions of Ag_0.025_LaFeO_3_ were examined via X-ray photoelectron spectroscopy XPS (Thermo ESCALAB 250xi, Waltham, MA, USA), where binding energy calibration was performed using the C 1s peak at 284.8 eV as the standard reference. Raman spectra were recorded on a Raman spectroscope (Renishaw InVia, Gloucestershire, UK) equipped with a laser excitation wavelength of 633 nm. Spectra were collected in the range of 100–800 cm^−1^ at room temperature under ambient atmosphere. Fourier-transform infrared spectroscopy test was recorded with a Fourier Transform Infrared Spectroscope (FTIR, Shimadzu IRAffinity-1S, Kyoto, Japan) in the range of 400–4000 cm^−1^ by the standard KBr pellet method at room temperature.

A semiconductor characterization system (4200A-SCS, Keithley, A Tektronix Company, Beaverton, OR, USA) was employed to characterize the resistive switching behavior of LaFeO_3_ and Ag_0.025_LaFeO_3_.

A Quantum Design SQUID vibrating sample magnetometer (SQUID-VSM, San Diego, CA, USA) was employed to investigate the magnetic characteristics of LaFeO_3_ and Ag_0.025_LaFeO_3_. The magnetic field-dependent magnetization (M-H) curves of the samples were acquired at 300 K.

## 4. Conclusions

Through electrospinning, this work not only achieved the synthesis of one-dimensional Ag-doped LaFeO_3_ NFs but also systematically elucidated their structural evolution, conduction behavior, and magnetic properties, further revealing the critical influence of Ag doping on the microstructure and multifunctional performance of LaFeO_3_ NFs. Rietveld fitting of the XRD patterns corroborated the orthorhombic crystal structure. The diameter values of LaFeO_3_ and Ag_0.025_LaFeO_3_ were about 160 and 100 nm, respectively. XPS results revealed that Fe exhibited a mixed oxidation state of +3 and +2, La remained predominantly in the +3 oxidation state, and O was mainly in the −2 state, with some contributions from oxygen vacancies and surface-adsorbed oxygen species. For Ag_0.025_LaFeO_3_ NFs, the conduction was dominated by the ohmic conduction mechanism in the LRS, while it was governed by space-charge-limited current conduction in the HRS. The Ag_0.025_LaFeO_3_ NF device showed a high on/off ratio, which improved the reliability of stored signals. It is thus indicative of the favorable prospect of Ag_0.025_LaFeO_3_ NFs for resistive random-access memory devices. The coercivity and remanence values of LaFeO_3_ were 150 Oe and 0.000494 emu g^−1^, while those of Ag_0.025_LaFeO_3_ were 200 Oe and 0.000404 emu g^−1^. This study demonstrates great application potential in magnetoresistive random-access memory devices.

## Figures and Tables

**Table 1:** Lattice parameters of LaFeO_3_ and Ag_0.025_LaFeO_3_.

Sample	*a* (Å)	*b* (Å)	*c* (Å)	*α*	*β*	*γ*	V(Å)^3^	Crystallite Size (nm)	GOF	*R_wp_* (%)	*χ* ^2^
LaFeO_3_	5.5720	5.5717	7.8614	90°	90°	90°	244.061	22	0.73	3.66	0.53
Ag_0.025_LaFeO_3_	5.5622	5.5933	7.8538	90°	90°	90°	244.340	23.9	0.80	3.83	0.64

GOF value represents the goodness of fit. *R_wp_* is the weighted reliable factor of the profile. ***χ*** is the Chi-squared value.

## Data Availability

The authors confirm that the data supporting the findings of this study are available within the article. In addition, the datasets used and/or analyzed during the current study are available from the corresponding author on reasonable request.
